# Myocarditis associated with COVID-19 vaccination

**DOI:** 10.1038/s41541-024-00893-1

**Published:** 2024-06-28

**Authors:** Alessandra Buoninfante, Arno Andeweg, Georgy Genov, Marco Cavaleri

**Affiliations:** 1 Public Health Threats, European Medicines Agency, Amsterdam, The Netherlands; 2 Pharmacovigilance Office, European Medicines Agency, Amsterdam, The Netherlands

**Keywords:** Vaccines, Pathogenesis, Epidemiology, Cardiovascular diseases

## Abstract

Following the start of the COVID-19 vaccination campaign, the adverse events of myocarditis and pericarditis were linked mainly to mRNA COVID-19 vaccines by the regulatory authorities worldwide. COVID-19 vaccines have been administered to several million people and the risk of myocarditis post COVID-19 vaccination has been characterised in great detail. At the present time the research data available are scarce and there is still no clear understanding of the biological mechanism/s responsible for this disease. This manuscript provides a concise overview of the epidemiology of myocarditis and the most prominent mechanistic insights in the pathophysiology of the disease. Most importantly it underscores the needed next steps in the research agenda required to characterize the pathophysiology of this disease post-COVID-19 vaccination. Finally, it shares our perspectives and considerations for public health.

## Introduction

The optimal response to a pandemic requires immediate and well-integrated action that includes, implementation of containment measurements, identification of the causative infectious agent, generation of specific diagnostics, and rapid development, authorization and roll-out of safe and effective therapeutics and vaccines. As demonstrated during the COVID-19 pandemic, prevention of disease by vaccination is crucial to reduce hospitalisation, mortality, and disruption of the healthcare system. Recently developed vaccine technologies like the messenger RNA (mRNA) vaccines proved to be most impactful since they paired short development and production timelines with high levels of protection. These vaccines have saved millions of lives and have been key in allowing the re-opening of society after a series of lockdowns.

Wide use of these vaccines was based on a clear positive benefit-risk balance, not only for the most vulnerable subjects prone to develop severe COVID-19, like the elderly and people with specific co-morbidities, but also for healthy individuals including those at young age. COVID-19 vaccines are very safe, nevertheless close safety monitoring post authorization resulted in the prompt identification of very rare adverse reaction events that were thoroughly assessed by EMA Pharmacovigilance Risk Assessment Committee (PRAC), and regulators worldwide. Among these, myocarditis and pericarditis cases emerged as very rare adverse events almost exclusively following immunisation with mRNA vaccines. These rare vaccination-associated disease manifestations could only be discovered post-authorisation since due to their low frequency they could not be identified earlier, not even in large trials involving tens of thousands of volunteers.

Pericarditis is a swelling and irritation of the tissue surrounding the heart (pericardium) and is usually mild and resolves without treatment. Myocarditis is an inflammation of the cardiac muscle (myocardium), which can reduce the ability of the heart to pump blood. In addition to immune activation due to e.g., autoimmunity and exposure to drugs (check-point inhibitors)^1^ these conditions have been linked to several viral infections^2^ including infection with SARS-CoV-2^3^ and also to vaccines, like live attenuated virus smallpox vaccines^4^. More recently pharmacovigilance surveillance and observational studies across the globe have pointed at an increased risk of myocarditis in predominantly younger males post administration of the two COVID-19 mRNA vaccines, Comirnaty and Spikevax, and also after vaccination with the adjuvanted protein-based vaccine Nuvaxovid and the adenovirus vector-based vaccine Jcovden^5–8^. Most cases of vaccine associated myocarditis are mild, transient, and self-limiting. However, occasionally, as with myocarditis caused by viral infection, chronic myocarditis and dilated cardiomyopathy might develop which can result in congestive heart failure and may even be fatal in very rare occasion^9^.

The efforts in collecting pharmacoepidemiology data to define incidence across populations are ongoing. The pathophysiology remains undefined and many disease mechanisms have been postulated^10,11^. The research data on the disease mechanism available are scarce and a definitive mechanism for the pathogenesis of this adverse event post vaccination is far from being established. In this manuscript we summarize the main pharmacoepidemiology data and highlight recent findings on the pathophysiology of COVID-19 vaccine associated myocarditis. We reflect on the information shared during an ad hoc workshop organized by the European Medicines Agency (EMA) and identify research areas important to improve our understanding of the pathophysiology of this very rare adverse event^12^. A better understanding of the disease mechanism leading to myocarditis upon vaccination is crucial for the design and use of improved next generation vaccines.

### Initial observations and regulatory actions

After the global COVID-19 vaccine rollout, the initial reports of a higher than expected number of myocarditis and pericarditis cases following mRNA vaccination emerged already in February–March 2021 from Israel^13^, Spain and the US. Within a few months also other countries reported increased rates (above the background incidence rate) of myocarditis and pericarditis after mRNA vaccination. The rapid identification by pharmacovigilance systems globally was followed by a prompt addition of a myocarditis and pericarditis warning to the product information of Comirnaty, Spikevax vaccines and later on, Nuvaxovid^5–7^ and Jcovden vaccines^8^. Data for Vaxzevria was reviewed by the PRAC and was considered insufficient to conclude on causal association, also noting that evidence from the literature was conflicting, with some studies^14–17^ showing a modest association whereas others did not^18–20^. For Spikevax and Comirnaty, the frequency of myocarditis and pericarditis has been listed as very rare ( < 1 in 10,000), while for Nuvaxovid and Jcovden it cannot be estimated from the data available so far, as more data are needed to further characterize the risk.

### Case definition

Identification of clinical manifestations of myocarditis is essential for accurate diagnosis and case management. In this context, the most used case definitions for myocarditis are taken from the US CDC^11^ and the Brighton Collaboration, and some epidemiological studies report on myocarditis by using the European Society of Cardiology diagnostic guidelines. These definitions largely overlap and all incorporate several types of evidence, such as clinical signs/symptoms, electrocardiogram, blood tests, imaging and histopathology, with the positive histopathological findings being regarded as gold standard for the diagnosis. Cardiac MRI is mostly used due its high sensitivity and non-invasive nature, however it is not being used very often for mild cases.

### Risk to develop myocarditis: COVID-19-vaccination vs COVID-19-infection

Before COVID vaccines were deployed and administered, it was reported that patients with COVID-19 have approximatively 16 times the risk for myocarditis relative to patients without COVID-19^3^_._ Matched analyses from medical records (Dec 2020 to May 2021) from the largest health care organization in Israel^21^ showed that COVID-19 vaccination was associated with an elevated risk of myocarditis (risk ratio, 3.24) compared to unvaccinated and SARS-CoV-2 infection was associated with a substantially increased risk of myocarditis (risk ratio, 18.28) compared to uninfected. It was also noted that a significant lower mortality rate was observed among individuals with myocarditis after mRNA vaccination when compared to those with a viral infection–related myocarditis^21,22^. In line with these results, a more recent study showed that the relative risk of heart failure within 90 days was 0.56 and 1.48 for myocarditis associated with vaccination and COVID-19 disease, respectively^23^. In summary, compared with myocarditis associated with COVID-19 disease, myocarditis after vaccination with SARS-CoV-2 mRNA vaccines occurs less frequent and in addition is associated with a better clinical outcome^23^. However, the accurate background incidence rate in healthy people and the actual number of vaccine associated cases are overall difficult to determine, and are most likely under-estimated, as many cases resolve without an actual diagnosis or assistance by healthcare providers and estimating the extent of potential under-reporting in the pharmacovigilance systems still remains a challenge as it may be influenced by numerous factors, including public awareness, the disease severity spectrum, demographics, and/or local practices.

### Risk to develop myocarditis upon COVID-19 vaccination: age and sex

At present, more than three years after licensing of the COVID-19 vaccines in the EU/ European Economic Area, it has been established that the highest risk of developing myocarditis is in males aged 12–30 years, within 1–14 days post vaccination after the second dose of the primary series of vaccination with an mRNA vaccine. Following post approval recognition of the increased risk to develop myocarditis upon mRNA COVID-19 vaccination, many large often nationwide or even global population studies revealed this risk distribution^14,16,24–30^. Two large European studies provided estimates of the number of excess cases of myocarditis after the second dose of mRNA vaccine in young vaccinees (below 30 years) compared to unexposed: 0.26 (French national health data system) and 0.57 (Nordic registry data) per 10,000 for Comirnaty, 1.3 and 1.9 per 10,000, respectively, for Spikevax^24^. For children aged 5–11 years, the US CDC has reported that myocarditis after mRNA COVID-19 vaccination is much rarer, with only slightly elevated rates above the anticipated background rates^31^. Importantly, myocarditis has not been reported among children aged 6 months–5 years^32^. Also for adults above 30 and the elderly, only slightly elevated myocarditis rates have been observed upon COVID-19 vaccination^2^. The US CDC has been following patients aged 12–29 years affected by myocarditis for at least 90 days after symptom onset and reported that for most of these the overall quality of life returned to pre-pandemic levels or were fully recovered as per health care provider assessment^33^. Of the 393 patients with biomarkers and/or imaging findings, 77–94% returned to normal or baseline values^22,31,33^.

It has been documented that classical viral infection–related myocarditis is more frequently observed in male than female individuals, from childhood through young adulthood, while women are affected mostly at the postmenopausal age^34^, which suggests that certain predisposing factors related to age and sex may contribute to the development of myocarditis post infection as well as to post-vaccination.

### Risk to develop myocarditis: vaccine dose interval and booster doses

The vaccine dosing interval could also impact the risk of developing myocarditis post vaccination. In this regard, a study from Canada demonstrated that the extended interval of 8 weeks as compared with a 3–4 weeks interval between mRNA vaccine doses 1 and 2 was associated with a reduced risk of myocarditis and pericarditis particularly among male individuals aged 18–24 years^25^. Furthermore, in Canada the highest reported incidence was observed among male adolescents aged 16–17 years after dose 2 (15.7 per 100,000) and the reporting rate was highest in those with a short (ie, ≤30 days) dose interval (21.3 per 100,000)^26^. Similarly, a French pre-print study indicated that for both the Comirnaty and the Spikevax vaccine longer intervals between each consecutive dose may decrease the occurrence of vaccine-associated myocarditis^35^ in line with a study performed in Hong-Kong also demonstrating that extended intervals between the first and second dose of the Cormirnaty vaccine reduces the risk of myocarditis with 29 percent in vaccinees aged 5–17 years^36^. In view of these data, some countries, i.e., Australia, Canada, UK, decided to extend the interval, 8–12 weeks, between primary course doses specifically for the very high risk group, 12–17 years old.

The risk to develop myocarditis upon administering mRNA booster doses has also been studied. Several studies have shown that the risk to develop myocarditis after a booster dose remains elevated but this risk is consistently lower than the risk after the second dose of the primary vaccination^27,29,37^.

It will be relevant to understand if this pattern holds true also with repeated booster vaccinations with updated COVID-19 vaccines.

### Relative risk to develop myocarditis for different COVID-19 vaccines

mRNA vaccines were the first COVID-19 vaccines to be authorized, distributed, and administered at an unprecedented large scale, while adeno-vectored and protein-based vaccines were authorized subsequently and administered at a smaller scale in the EU/European Economic Area. Initial meta-analysis comparing the risk associated to immunization with these respective vaccines suggested a higher incidence of myocarditis after receiving an mRNA vaccine vs a non-mRNA (adeno-vectored) vaccine^2^, however this analysis should be updated with data including more recently authorized vaccines. Among the mRNA vaccines, individual studies suggested a higher incidence of myocarditis after a second dose of Spikevax compared to Comirnaty^16,38^.

### Full disease spectrum of COVID-19 vaccine associated myocarditis captured?

Studies on myocarditis associated with COVID-19 vaccination are hampered by several limitations such as: the absence of reliable estimates of the clinically suspected myocarditis frequency in the population prior to COVID19 pandemic, as well as lack of a systematic collection of endomyocardial biopsies to support the confirmation of the diagnosis, especially for clinically mild cases in which collecting biopsies is not warranted. Of note, usually biopsies are not taken, and MRI is not performed in clinically mild cases.

In a study specifically including recently (≤20 days) COVID-19 vaccinated individuals that died unexpectedly^9^, it was shown that most of these cases displayed an inflammatory infiltration of the epicardium and the subepicardial fat tissue. This infiltrate was characterized by an identical T cell-dominant immunophenotype and interspersed macrophages. An acute arrhythmogenic cardiac failure was postulated due to the limited pathology observed. No causal association with COVID-19 vaccination can be asserted from this study, but the authors suggested that CD4 + T cells are the main drivers of heart-specific autoimmunity in myocarditis^39^. Additional research is required to assess the relevance of the results obtained.

## Mechanistic insights in the pathophysiology of COVID-19 vaccination associated myocarditis

**Fig. 1:**
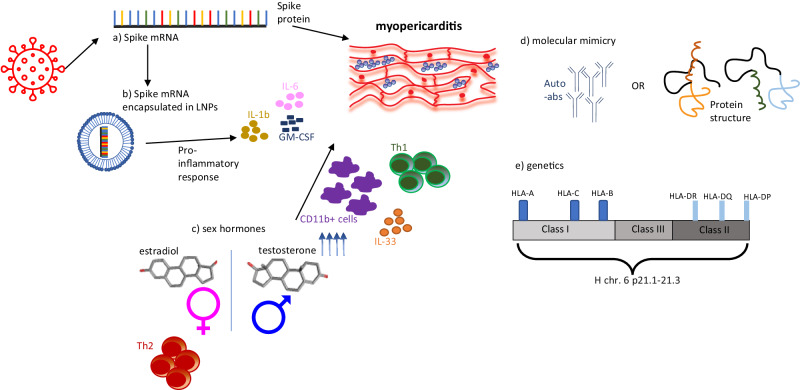
Summarizing overview of the potential mechanisms of myopericarditis following COVID-19 vaccination. **a** The spike sequence introduced in the COVID-19 vaccines is the antigen recognized by the immune system, which activate specific immune responses, some of which could directly contribute to inflammation of the endothelium and cardiac tissue. **b** In the mRNA vaccines, the spike mRNA sequence is encapsulated in lipid nanoparticles (LNPs) for the delivery. The LNPs can elicit a variety of cytokines and chemokines, which can trigger a proinflammatory response with impact on the myo/pericardium. **c** Vaccine myocarditis is present mostly in young men, which suggests a role for sex hormones. While estradiol activates a T helper cell (Th) 2 response and anti-inflammatory cytokines, testosterone activates pro-inflammatory Th1 responses. **d** As myocarditis can be driven by autoimmune responses, molecular mimicry between spike protein and antigens associated with myocarditis in terms of protein sequence and structure could lead to myopericarditis by triggering cross-reactivity. **e** Genetic factors, such as specific HLA genes, may contribute to the risk to develop myocarditis upon mRNA vaccination as seen with viral infection associated myocarditis. The 3D structure image of estradiol, PubChem Identifier: *CID 5757*, was taken from URL: https://pubchem.ncbi.nlm.nih.gov/compound/Estradiol#section=3D-Conformer. The 3D structure image of testosterone, PubChem Identifier: *CID 6013*, was taken from https://pubchem.ncbi.nlm.nih.gov/compound/Testosterone#section=3D-Conformer.

Many hypotheses have been advanced regarding the etiology of myocarditis post COVID-19 vaccination (Fig. 1). Most of these relate to hyperimmunity or dysregulated immune responses and their triggering and modifying factors ultimately resulting in myocardial inflammation. Unfortunately, a biomarker associated with COVID-19 vaccine related myocarditis nor disease mechanism for vaccine induced myocarditis has been identified, which may inform the development of next generation mRNA vaccines.

### Role of IL-1 RA

It has been shown previously that interleukin 1 (IL-1) and interleukin 1 receptor antagonist (IL-1ra) are key regulators of the immune response elicited by mRNA vaccines; the IL-1 pathway plays a key role in triggering RNA vaccine-associated innate signaling^40^ in mice, non-human primates and in humans. In addition, neutralizing autoantibodies targeting the endogenous IL-1RA, which inhibit interleukin-1 signaling and inflammation, have been found in adults with critical COVID-19 and also in cases of multisystem inflammatory syndrome in children^41,42^. Interestingly in a study including patients with histologically confirmed myocarditis, the authors detected anti–IL-1RA antibodies in 75% of patients younger than 21 years of age, as compared with 11% of 21 years of age or older^43^. Furthermore, anti–IL-1RA antibodies were not detected in the remaining patients in whom biopsy ruled out the diagnosis of myocarditis and only in 1% of the control samples. At the time of acute myocarditis, IL-1RA plasma levels correlated with markers of cardiac damage (troponin T, creatine kinase, creatine kinase MB, or pro–B-type natriuretic peptide), the cardiac-tissue infiltration of CD3 + T cells and CD68+ macrophages, and also with systemic inflammation (C-reactive protein), while there was a negative correlation between markers of cardiac damage and IL-1RA plasma levels in patients with anti–IL-1RA antibodies.

Preliminary data suggest that a transient hyperphosphorylation of IL-1RA at specific sites precedes a breakdown of peripheral immune tolerance and consequent induction of anti-IL-1Ra-autoantibodies. Of note, IL-1RA autoantibodies were not found in a small cohort of 8 myocarditis patients post Comirnaty. In this cohort, however, increased frequencies of activated cytotoxic CD8 T cells and NK cells were measured, which suggest the role of innate immune activation in vaccine-associated myocarditis^44^.

In this context, while differences between humans and rodents are acknowledged, it will be interesting to study the role of IL1-RA autoantibodies in animal models of myocarditis and assess the impact of the break-of-tolerance on the course of the disease, the role of mRNA vaccine formulation on IL-1RA hyperphosphorylation, and the presence of phosphorylated IL-1RA in cardiac tissue. This could improve our understanding of the role of IL1-RA and its associated hyperphosphorylation in vaccine induced myocarditis and its potential use as a biomarker of myocarditis post vaccination.

### Contribution of innate immunity and cytokines

In a case of a patient affected by myocarditis post COVID-19 mRNA vaccination, cytokines were detected in plasma on day 3 post vaccination, especially elevated levels of IL-18 and activated NK cells and T cells were found, while healthy vaccinated controls did not show an equivalent increase^45^. Follow-up experiments in mice showed that in vivo IL-18 administration caused NK cell and T cell activation in the heart and mild cardiac dysfunction. Thus, a possible mechanistic explanation for myopericarditis after COVID-19 mRNA vaccine could be cardiac injury mediated by vaccination-triggered excessive IL-18 production and strong NK and T cells activation. In addition to an elevated IL-18 production, the patient with myopericarditis following COVID-19 mRNA vaccination had increased monocytes numbers, which are a primary source of IL-18 in the blood^46^. The investigators could not detect any spike protein in the endomyocardial biopsy of the patient, as well as no significant T cell infiltration in the myocardium. Monocytes and macrophages are the main inflammatory cell subsets found in natural and experimentally induced myocarditis^47^. Some investigators have observed eosinophils within mixed inflammatory infiltration in endomyocardial biopsy and autopsy specimens of patients with myocarditis after COVID-19 mRNA vaccination^48^, which is less typical than most cases reported. Indeed, in a study conducted in young male patients who developed myopericarditis shortly after SARS-COV-2 mRNA vaccination, eosinophilic myocarditis was not observed^49^. Of note, unbiased serological analyses of these patients revealed elevated levels of IL15, IL-1β and IL1-RA, additionally to various matrix metalloproteases and chemokines, activated cytotoxic T lymphocytes and monocytic dysregulation^49^.

As the timing of sampling is crucial to detect the stage of the disease progression, the distinct study results including cytokine responses and the exact nature of lymphocytic infiltrates could relate to differences in timing of sampling across studies. Also, it stresses the need to perform more detailed kinetic analysis of the immune responses, as these are dynamic over time. Most of the data collected so far seem to point at a major contribution of the innate immune system potentially shaping the nature of the adaptive system in myocarditis post COVID-19 vaccination. Regarding the mRNA vaccine platform technology, to achieve an effective and targeted immune response and to avoid major local and systemic adverse events, a tight regulation of the two branches of the immune system is required. At present it is still unknown which innate immune pathways may contribute to protective immunity and which one to the reactogenicity^50^. Most of the innate immune activation seems to rely on the lipid nanoparticles containing ionizable lipids component of mRNA vaccines, as this component can elicit a variety of cytokines including IL-1β, IL-6, GM-CSF, and many chemokines. It has been shown in animal models that particularly IL1 is a key mediator of the reactogenicity profiles of mRNA vaccines^40^. Dissecting the molecular mechanisms by which mRNA and lipid nanoparticles are sensed may contribute to the development of less reactogenic and possibly even more effective vaccines.

Studies focusing on specific (classes of) immune mediators like cytokines and chemokines, or specific immune cell populations are disclosing important information about the (dys)regulation of immune responses underlying or associated with vaccine induced myocarditis. Integration of the results obtained with separate studies e.g., those pointing at changes in IL-1 and IL-18 signaling pathways however pose a challenge due to the complexity of the immune system. Systems vaccinology^51^, the application of a range of -omics technologies and bioinformatics approaches in vaccine research, allowing “genome wide” studies on immune processes could support data integration, interpretation as well as the generation of new hypothesis^52–54^. So far, omics studies have mostly used serum or blood (cell) samples, complementing these studies with single cell sequencing approaches using locally sampled cells as input is expected to provide relevant information on the pathophysiology of vaccine associated myocarditis.

### Implications for free spike protein

Despite not being systematically screened and checked for in myocarditis samples, the spike protein could be a key player in the etiology and/or progression of the disease. Particularly it has been speculated that the spike protein (expression) could directly contribute to inflammation of the endothelium and cardiac tissue. Detailed antibody- and T-cell multiplex cytokine response profiles of 16 subjects who developed postvaccine myocarditis within the first week after vaccination appeared to be indistinguishable from those generated from vaccinated control subjects and only a modest increase in cytokine production linked to innate cell response was observed. However, elevated levels of full-length spike protein unbound by antibodies were detected in the plasma of these myocarditis subjects, whereas no free spike was detected in asymptomatic vaccinated control subjects^55^. Interestingly, the unbound spike correlated with cardiac troponin T levels and innate immune activation with cytokine release. In vitro data have shown that the spike protein can interact with and cause dysfunction of cardiac pericytes^56^ and contributes to endothelial inflammation in mice^57^. These findings are in line with data^58,59^ demonstrating the presence of spike antigen post second COVID-19 mRNA dose in circulation longer than originally anticipated. The data of Yonker et al. may point to a direct role of the unbound circulating spike protein, not in inducing immune hyperactivation, but in contributing directly to the effects recorded in myocarditis subjects. Additionally, some have hypothesised that the inadvertently intravenous injection of COVID-19 mRNA vaccines may induce myopericarditis, as suggested by the histopathological changes, mRNA expression, and serum levels of cytokine/chemokine and troponin in Balb/c mice injected with Comirnaty^60^. Histopathological changes in these mice became more diffuse after a second vaccine injection. The authors found accumulation of unfolded spike protein at the endoplasmic reticulum, which, if in excess, can activate apoptosis.

### Molecular mimicry and autoimmunity

As myocarditis can be driven by autoimmune responses, it was also examined if the spike protein was potentially similar to autoantigens previously identified to be associated with myocarditis. This was assessed by sequence identity comparison analysis between the spike protein-derived peptides and myocarditis-associated antigens, and by structural analysis of these antigens and the spike to identify potential discontinuous 3-D epitope similarities^61^. A set of approximately 40 different antigens was considered. However, the study did not show a significant enrichment in the frequency of spike-derived peptides similar to myocarditis-associated autoantigens as compared to controls. It must be stressed that the lack of evidence does not necessarily reject completely the molecular mimicry hypothesis, additional experimental studies need to be performed to fully exclude the possibility of spike protein induced autoimmunity.

### Sex and hormonal differences

The immunological response in classical myocarditis is significantly different between males and females^62^. Females have low levels of cardiac inflammation during myocarditis due to an elevated anti-inflammatory immune response that is characterized by higher numbers of Th2-type immune cells, an anti-inflammatory macrophage phenotype, greater numbers of Foxp3+ regulatory T cells, and higher overall levels of anti-inflammatory cytokines. In contrast, males produce a robust proinflammatory immune response during myocarditis that is characterized by mast cell activation, macrophage response that promotes elevated proinflammatory cytokines and increased complement pathways. This leads to the inhibition of the regulation of the inflammatory response which explains the distinct severity level of inflammatory heart disease between the sexes. Moreover, in men affected by myocarditis and in male mice with coxsackievirus B3 (CVB3) infection induced myocarditis, the myocardial infiltrates found were predominantly CD11b positive^63,64^. Men with myocarditis have worse recovery outcome and a higher need for transplantation compared with women.

Besides the intrinsic immunological differences between males and females, difference in the disease prevalence and outcome between the sexes have been linked to hormones^65^. In a clinal study of myocarditis, a biomarker of heart failure released in response to vascular congestion and inflammatory and pro-fibrotic stimuli, soluble suppression of tumorigenesis-2 (sST2), was found increased in the sera of male subjects below 50 years of age but not in females. This biomarker correlated with myocardial inflammation and poor heart function, and it was increased by testosterone but not estradiol in mice with myocarditis, which would suggest that in men sST2 is increased because of testosterone levels. Of note, sSt2 plays a crucial role in cardiac inflammation via IL-33 on innate immune cells, which then trigger expression of Cd11b + , which as described above is highly expressed in biopsies of males with myocarditis. Also, testosterone itself contributes to increasing levels of Cd11b and other pro-inflammatory cytokines, such as IL-1β, TLR-4 and caspase 1^62^.

Finally genetic factors, related to sex or not, may also contribute to the risk to develop myocarditis upon mRNA vaccination like seen with viral infection associated myocarditis. Myocarditis resulting from an enterovirus or human herpesvirus infection, which is tendentially more severe with younger age and male sex, has been associated with an immune–genetic background in genes encoding HLA factors^11^. However, the mechanism is still unclear and the attempts so far of analysing individual or few samples have only provided limited and inconclusive information. Despite the difficulty in obtaining samples pre and post-myocarditis, efforts are ongoing in performing unbiased approach analyses^66^, including genome-wide association studies. This could be a promising area of research, as HLA alleles have been associated with adverse events following the administration of vaccines: HLA-A∗03:01 carrying individuals are predisposed to more pronounced fever, chills, and stronger side effects from Comirnaty vaccination^67^.

## Concluding remarks

Available evidence shows that COVID-19 mRNA vaccination is associated with an increased risk of myocarditis, but at a much lower level than the risk associated with COVID-19 infection, reiterating a clear positive benefit/risk ratio for COVID-19 mRNA vaccines. To date no definite mechanism for vaccine associated myocarditis has been identified and additional epidemiological, clinical and non-clinical research is required. The EMA will continuously monitor the emerging evidence and update the public and the product information of COVID-19 vaccines as necessary. The key objectives to fill in the knowledge gaps were summarised in a research agenda presented here below and in Table 1.

**Table 1:** Tabular overview of the research agenda for myopericarditis

Area	Objective
Pharmacovigilance	Study the long-term prognosis of subjects who experienced vaccine associated myocarditis
Pharmacovigilance	Characterize the risk of myocarditis after more booster doses and ascertain the link with timing of vaccine doses administration
Pharmacovigilance	Collect and analyse data from the use of other type of vaccines than mRNA to ascertain the potential association with myo-pericarditis
Clinical	Establish large and high quality myocarditis patients cohort
Clinical	Assess the existing registries and conduct post-mortem examination to identify potential differences between true and self-limiting cases
Clinical	Characterize the role interplayed between the two branches of the immune system
Clinical	Identify and validate biomarkers of myocarditis in human samples
Clinical	Search for possible genetic risk factors via omics approaches
Clinical	Assess the contribution of the spike protein for the trigger or progression of myocarditis
Non-clinical	Establishing and optimizing vaccine and/or viral infection induced myocarditis animal models
Non-clinical	Evaluate the impact of the break of immune tolerance in the course of the myocarditis disease in animal models

### Research on epidemiology

Pharmacovigilance surveillance and observational studies have identified an increased risk of myocarditis predominantly in younger males post mRNA vaccination. It has been shown that increasing the time interval between mRNA vaccine doses decreases the risk of vaccine-induced myocarditis^25,35^. In this context, it will be relevant to characterize the risk of myocarditis after multiple mRNA booster doses and ascertain the link with timing of vaccine doses administration. Ideally this research should not only include vaccine associated myocarditis patients but also background myocarditis patients to provide the optimal context. Epidemiology studies on possible long-term adverse effects of vaccine associated myocarditis are required. The marketing authorization holders are planning clinical studies to collect long-term primary data over a 5-year period with the aim to further evaluate the clinical course, risk factors, long-term sequelae, and quality of life of post-vaccine myocarditis/pericarditis^68,69^. To have a more comprehensive overview, also public health authorities and academic groups should join their effort in studying the long-term prognosis of subjects that experience vaccine-induced myocarditis. Furthermore, it would be desirable to collect more evidence from the use of other type of vaccines than mRNA, such as adjuvanted-protein based vaccines, to ascertain whether also these vaccines can be associated with myopericarditis and exclude the possibility of a single platform bias.

### Clinical research

As emerged from the clinical studies cited in this review and the case reports/series available on non-invasive investigations and biopsy findings, the reported pathological patterns of vaccine associated myocarditis varies greatly. The different tissues and blood sampling schedules applied could be an important factor influencing the already complex disease scenario. Furthermore, the question remains open on whether the myocarditis cases post SARS-COV-2 infection are triggered by a similar mechanism than the cases recorded post COVID-19 mRNA vaccination or not. Therefore, establishing high quality patient cohort(s) enabling the standardized collection of relevant time course samples supporting a wide range of exploratory and confirmatory studies would be one of the starting points. On the other hand, the assessment of the existing registries, post-mortem examination, and performance of autopsy of rare lethal cases of myocarditis, is also important to identify potential differences with self-limiting cases and to further obtain insights in the pathophysiology of myocarditis and discern true cases from other similar diseases. Most of the data collected so far seems to point at a major contribution of the innate immune system compared to the adaptive system in myocarditis post COVID-19 vaccination. Additional studies should aim to better characterize the role interplayed between the two branches of the immune system in these patients. Exploring the distinct features of the mRNA vaccine platform could provide insights for the development of less reactogenic vaccines. Lastly, the presence of the spike protein in patients suffering from myopericarditis should be investigated in larger studies that could measure simultaneously the presence of biomarkers of inflammation and immune cell profiles. At the present time it is unclear if and to what extent the spike protein plays a role in the etiology of this adverse event after mRNA vaccination. Clarification on this aspect would have significant impact on vaccine design for any future generation of COVID-19 vaccines. Discerning the contribution of the mRNA platform to myocarditis has also important implications with respect to other vaccines that will be developed with this technology.

### Nonclinical research

Regarding the non-clinical research, the focus should be on establishing and optimizing vaccine and viral infection induced myocarditis animal models, as these need to mimic human immunity as closely as possible. These models could be used to explore several lines of investigation including the impact of the break of immune tolerance. Based on the preliminary results obtained by Thurner et al. it seems relevant to investigate also the anti-IL1RA autoantibody responses in the CVB3 animal model, as these antibodies were preferentially detected in patients with suspected myocarditis post COVID-19 vaccination^43^. In addition to this specific example animal myocarditis models will also enable studies on the role of vaccination triggered or dysregulated innate (or adaptive) immune responses that may result in or modify myocardial inflammation. Animal models will also be instrumental in dissecting the postulated role of the mRNA vaccine platform as such (lipid nanoparticles) from the potential role of the expressed pathogen derived antigen(s) in triggering myocarditis. In the framework of COVID-19 mRNA vaccine development the SARS-CoV-2 spike protein should be evaluated but any other target antigen can be analyzed as well. Myocarditis animal models^70^ could provide valuable information for the development of any other future mRNA platform based vaccine.

### Implications for public health

The challenges in addressing the above-mentioned research questions are acknowledged. Clarifying those questions would however allow for measures to minimize any potential risk with current vaccines and would offer the possibility to consider adjustments in vaccines design to further mitigate such risk. The mRNA platform is an important addition to the armamentarium of technologies for future vaccines which demonstrated safety and efficacy in the context of COVID-19. In light of the expected continued use for re-vaccination campaigns, any adjustment that could reduce the risk of myopericarditis and thus boost confidence in these vaccines would be welcome. Finally, responding to the afore-mentioned questions would give insights into the mRNA vaccine platform with implications not only for the future design of vaccines against newly emerging infectious diseases but also for a myriad of other areas of medical application.
